# Hippocampal Neurosustainability for Stress Resilience: A Pro-Neurogenic BDNF-Targeted Architectural Enrichment Framework to Overcome Type 2 Allostatic Overload

**DOI:** 10.3390/brainsci16040370

**Published:** 2026-03-29

**Authors:** Mohamed Hesham Khalil

**Affiliations:** Department of Architecture, University of Cambridge, Cambridge CB2 1PX, UK; mhmhk2@cam.ac.uk

**Keywords:** hippocampus, adult hippocampal neurogenesis, BDNF, chronic stress, resilience, environmental enrichment, environmental stressors, neurobiophilia, stair use, physical activity

## Abstract

**Highlights:**

**What are the main findings?**

- Urban living is associated with heightened stress and inactivity that together form type 2 allostatic overload, but failing to recover from stress is primarily due to unsupported neurogenesis.
- Adult hippocampal neurogenesis can inhibit HPA-axis overreactivity and enhance the dentate gyrus pattern separation ability towards stressors.
- Architectural enrichment through neurobiophilic design and stair use may sustainably elevate BDNF whilst mitigating stress to enhance adult hippocampal neurogenesis in humans, ultimately supporting long-term stress resilience.

**What are the implications of the main findings?**

- Architectural enrichment can capitalise on the extensive time spent indoors, such as at home or in the workplace, to improve the brain’s ability to overcome the daily stressors associated with urban living.

**Abstract:**

Chronic stress is among the most pervasive health challenges of contemporary urban life, yet its persistence is not simply a matter of external pressure. When adult hippocampal neurogenesis is impaired, the brain loses its capacity to regulate the hypothalamic–pituitary–adrenal (HPA) axis and distinguish new threats from familiar ones through dentate gyrus pattern separation, rendering stress self-perpetuating. Physical activity is widely recognised as a promoter of neurogenesis through brain-derived neurotrophic factor (BDNF), yet the built environments in which most people spend approximately 90% of their time simultaneously suppress BDNF through chronic stress and deny sufficient physical activity intensity to restore it, a condition known as type 2 allostatic overload sustained by architectural impoverishment. This paper proposes architectural enrichment as a theoretical framework designed to resolve this problem at its root through two independent but synergistic mechanisms: architecturally mediated voluntary stair use to elevate peripheral BDNF via metabolic pathways, and neurobiophilic design based on the Neurobiophilia Index to attenuate cortisol and passively support BDNF and neurogenesis. Twelve hypothesised neurobiological profiles are derived in a framework that advances the concept of hippocampal neurosustainability, proposing that buildings can be designed not merely to avoid harming the brain but to actively sustain its capacity for resilience amid the stressors of modern urban living.

## 1. Introduction

Chronic stress is arguably one of the most pervasive health challenges of the twenty-first century [1,2,3], but the real problem lies in the impairment of adult hippocampal neurogenesis, which prevents the brain from recovering from stress [4,5,6]. Chronic stress turns into allostatic overload when it is combined with sedentary behaviours [7,8,9,10], which become associated with poorer mental and physical health [11], while sustained physical activity is itself a sufficient promoter of adult hippocampal neurogenesis [12,13] through a mechanism largely associated with brain-derived neurotrophic factor (BDNF) [14].

After it was hypothesised that allostatic overload is highly associated with urban lifestyles due to elevated stress and physical inactivity [15], subsequent neuroscience evidence strongly supports this hypothesis. For instance, after acute exposure to stress, a walk in the forest, but not in an urban street, reduced amygdala activity [16] and increased the subiculum volume [17], which functions as an inhibitory regulator of the hypothalamic–pituitary–adrenal (HPA) axis [18]. Further, urban grey space is associated with increased default mode network (DMN)-amygdala connectivity that reflects emotional dysregulation [19]. Nonetheless, even moderate-intensity cycling activity in an air-filtered indoor environment can increase BDNF compared to similar activity near a major traffic road with air pollution [20], while walking in a temperate environment fails to increase BDNF compared to walking in a hot environment [21]. Together, as urban living fails to provide stress recovery and its infrastructure may not be sufficient for elevating BDNF [22], it can pose a great risk of allostatic overload and may not be sufficient to support the adult hippocampal neurogenesis that is necessary for stress resilience.

In contrast, the extensive time spent inside buildings, such as the home or the workplace, which is estimated to be around 90% of the time [23], can be an opportunity to increase BDNF and likely sustain adult hippocampal neurogenesis. Hence, the framework established in this paper by the author is established after earlier published works, including the theory of neurosustainability [24], which is the sustainability of adaptive plasticity in humans through environmental enrichment, and the architectural enrichment tools targeting BDNF with a focus on stress and physical activity among other factors [25,26]. First, the environmental affordance for physical activity and its application through staircases in buildings suggests that stair use can elevate BDNF [25]. Second, the Neurobiophilia Index proposes that BDNF and neurogenesis can be elevated and sustained, respectively, through a number of items scored on a symmetrical scale, as Neurobiophilia is based on how nature nurtures the brain [26]. Together, this paper establishes a framework that aims to achieve hippocampal neurosustainability for stress resilience towards urban living.

The framework in this paper is promising and permits a cross-species analysis of evidence since adult hippocampal neurogenesis, despite earlier contradictory results in early studies on humans [27,28,29], has been proven to persist in humans into the tenth decade of life [30,31,32,33,34], and undetectability has been attributed to methods of study [35]. Its decline in human neurogenesis is associated with increased Alzheimer’s disease risk [30,31,34] and is explained by a reduction in *BDNF* gene expression [34], while in laboratory models, hippocampal *Bdnf* gene expression primarily increases through voluntary physical activity [36]. Adult hippocampal neurogenesis in both rodents and humans is convergent in biological mechanisms, including the BDNF-Trkb binding triggering of downstream Akt that promotes cell proliferation and differentiation [37,38]. Together, this paper confidently establishes a theoretical basis for its framework.

## 2. Adult Neurogenesis, BDNF, and Stress Resilience

The relationship between stress and adult hippocampal neurogenesis is bidirectional, largely mediated by BDNF, and must be explained through both animal and human studies to understand the full dynamics due to the convergent neurogenesis-based biological processes and the insufficiency of human-based evidence.

First and foremost, it is important to review the commonly shared neurobiological mechanisms between humans and rodents regarding neurogenesis before synthesising evidence on stress, BDNF, and adult hippocampal neurogenesis conducted across different species. As adult hippocampal neurogenesis persists in both humans and rodents [39,40,41], some convergent biological mechanisms can help synthesise evidence on both subjects. Zhou et al. [38] reported in their study that immature dentate granule cells across humans, macaques, pigs, and mice converge on shared biological processes, including neurogenesis, despite the divergent use of genes to achieve it. Charou et al. [37] explained in their study that the BDNF-TrkB signalling pathway shows notable conservation across species, triggering downstream Akt and promoting cell proliferation and differentiation.

Disouky et al. [34] showed in their study that *BDNF* gene expression in humans is associated with adult hippocampal neurogenesis. Understanding the relationship between physical activity and brain-derived neurotrophic factor in humans remains exclusive to peripheral BDNF in serum or plasma, while the same relationship is explained at the level of *Bdnf* gene expression for rodents. On the one hand, a plethora of studies support that acute moderate-to-vigorous-intensity physical activity can increase serum or plasma BDNF in humans. For instance, a 20 min gardening activity with a metabolic equivalence (a unit used to estimate the energy expenditure and intensity of physical activities) of 3.5 classified as moderate-intensity [42], 6 min of high-intensity interval training (HIIT) cycling [43], a 30 min moderate-intensity walk [44], a 180 min walk in 32 degrees Celsius [21], and a 5 h golf walk [45] were all able to increase peripheral BDNF in humans. On the other hand, physical activity through the use of running wheels has been associated with *Bdnf* transcripts and gene expression [46,47,48], *Bdnf* gene expression, and activation in BDNF-TrkB binding in rodents [49,50]. Despite the gap between physical activity and *BDNF* gene expression in humans persisting, the commonly shared BDNF-TrkB signalling pathways between rodents and humans regarding adult hippocampal neurogenesis make the hypothesis that physical activity supports *BDNF* gene expression and neurogenesis in humans plausible.

Studies performed on rodents suggest that the relationship between stress and adult hippocampal neurogenesis is bidirectional, largely explained by its effect on *Bdnf* expression. First, a well-established consequence of chronic stress is the suppression of brain-derived neurotrophic factor and TrkB [51,52], which is central to adult hippocampal neurogenesis [53]. Adult hippocampal neurogenesis is essential for regulating the HPA axis in response to stress [54,55,56,57] and for discriminating between novel and familiar stressors through the enhanced pattern separation ability of the dentate gyrus [6,58,59]. Second, while hippocampal *Bdnf* expression plays a critical role in resilience to chronic stress [60], chronic stress reduces *Bdnf* mRNA levels in the rat hippocampus, but the effect appears weaker compared to acute stress [51].

In humans, despite no evidence that shows how stress affects *BDNF* gene expression, chronic stress is associated with lower basal serum BDNF levels, and while acute stress increases both serum BDNF and cortisol, stress-induced higher cortisol secretion can accelerate post-stress BDNF decline, suggesting it can reduce neurogenesis in humans [61]. The acute stress–BDNF dynamics, therefore, support the results derived from rodent studies, and they suggest that the long-term benefits of increasing BDNF in humans may provide stress recovery and resilience in humans as they do for rodents.

## 3. Architectural Enrichment: A Theoretical Framework for Stress Resilience

Allostatic overload needs to be understood in context to resolve the problem, as urban living is closely associated with a particular type of allostatic overload [15]. Environmental stressors can force the body into a sustained state of adaptation, causing cumulative physiological wear and tear, which, when these demands exceed the body’s ability to cope, leads to a state called allostatic overload [61,62]. While type 1 allostatic overload occurs when energy demands exceed energy supply, such as in extreme environmental conditions or critical illness, type 2 allostatic load is closely tied to modern urban living and the habits that come with it. It is defined by two key features: consuming more calories than the body burns through physical activity and being regularly exposed to mild but ongoing predictable sources of stress [15].

**Figure 1:**
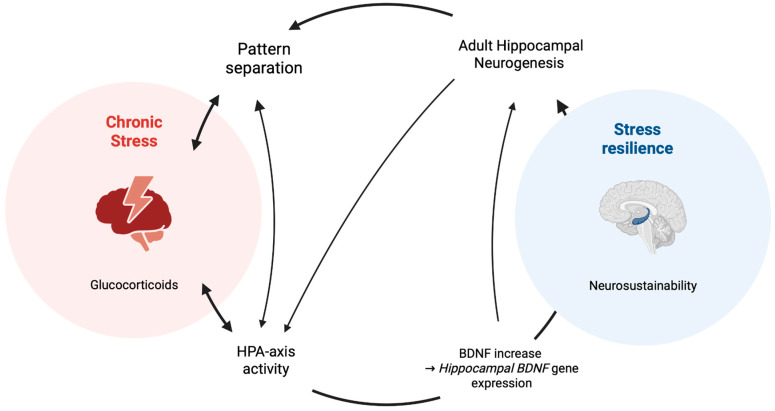
Proposed relationships between chronic stress, adult hippocampal neurogenesis, and stress resilience. Chronic stress is hypothesised to dysregulate the HPA axis and elevate glucocorticoid levels, which in turn suppress adult hippocampal neurogenesis (AHN) and impair pattern separation of the dentate gyrus. This relationship is further hypothesised to be bidirectional, whereby diminished pattern separation capacity reinforces HPA-axis hyperactivation, perpetuating a stress-vulnerability cycle. AHN is proposed as a central mechanistic node linking chronic stress to stress resilience, with sufficient neurogenic output hypothesised to restore adaptive pattern separation and attenuate HPA-axis reactivity. Finally, increases in hippocampal BDNF gene expression are hypothesised to drive AHN and promote hippocampal neurosustainability, ultimately facilitating the transition from chronic stress vulnerability to stress resilience.

**Figure 2:**
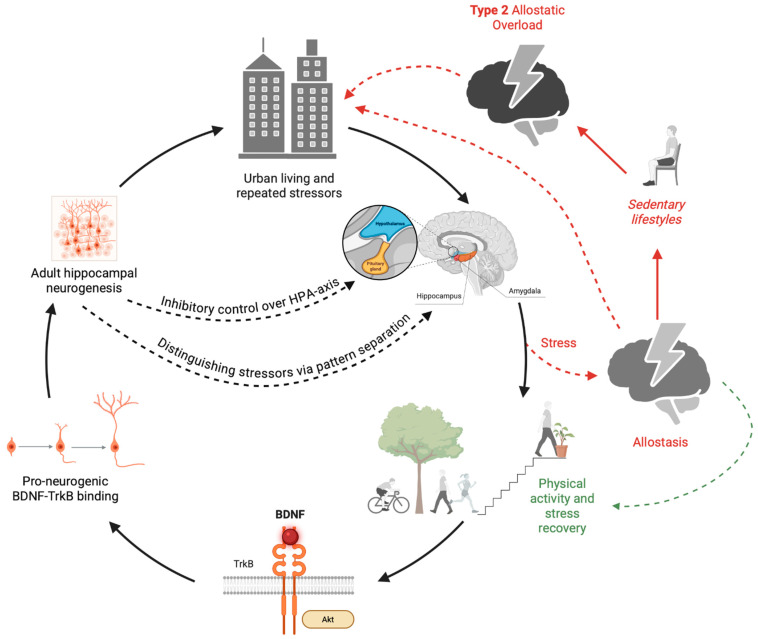
The figure illustrates that the human brain can live within the red-arrow cycle, where stress becomes chronic, or within the black-arrow cycle, where the brain can recover from stress. A pro-neurogenic environmental enrichment framework for stress resilience in urban environments through architecturally mediated physical activity, neurobiophilia, and improved urban infrastructure. Successful enrichment targets a sustainable elevation in BDNF that binds to TrkB in humans to trigger downstream Akt and promote cell proliferation and differentiation to ultimately achieve hippocampal neurosustainability. A sustainable support to adult hippocampal neurogenesis can provide stress recovery and resilience through the control of the HPA-axis and the enhanced dentate gyrus pattern separation ability towards repeated psychosocial stressors.

This novel framework, architectural enrichment, acts as an antidote to the prevalent type 2 allostatic overload in contemporary lifestyles through addressing the two root causes of the problem: BDNF-targeted architecturally mediated voluntary physical activity to sustainably counteract sedentary behaviours and BDNF-regarded neurobiophilic design for stress recovery. To contextualise the architectural enrichment framework for stress recovery and resilience, this paper treats it as an opposite pole of type 2 allostatic overload, where there is a lack of physical activity opportunities sufficient to increase BDNF and a lack of stress-recovery opportunities. Each mechanism, however, is independent and may be embodied separately or synchronously in the architectural space. Figure 1 illustrates the foundation of the architectural enrichment for the stress-recovery and resilience framework, while Figure 2 illustrates the feedback loop between the built environment, stress, and neurogenesis.

### 3.1. Architecturally Mediated Physical Activity and BDNF

To understand how architectural enrichment elevates peripheral BDNF concentrations in humans, it is important to understand that BDNF concentrations may change in response to voluntary physical activity that is intensity-dependent. To determine how a built environment can have the potential to increase BDNF, metabolic equivalents (METs) were used to explain this relationship [63]. This is supported by research demonstrating that a 20 min gardening activity with a 3.5 MET value classified as moderate-intensity [42], 6 min of HIIT cycling [43], and a 30 min moderate-intensity walk [44] are all sufficient to elevate peripheral BDNF.

In buildings, indoor walking is very unlikely to reach moderate intensity, as the 2024 Adult Compendium of Physical Activity shows that indoor activities are very unlikely to reach 3 METs, but stair use is generally moderate-to-vigorous intensity (>3 METs) [64]. Staircase design and stair use were modelled in a previous paper as potential drivers for stair use activity intensity in buildings to the extent that they may elevate BDNF [25]. Although effective HIIT-based stair use is seen as more demanding than the exercise snacking approach (single ascent bouts spread across the day) [25], despite the fact that exercise snacking is preferred over HIIT regarding stair use, even though both have similar positive affective outcomes [65], it is very unlikely that it can impair the increase in BDNF. For instance, cycling indoors was effective in elevating BDNF, unlike cycling outdoors amid air pollution [20], which demonstrates that architectural stressors, such as geometrical features [66], may be very unlikely to cause significant impairment in BDNF increase.

In addition, whether voluntary or involuntary stair leads to different BDNF concentration changes, but the difference is very unlikely to prevent an increase in BDNF. For instance, in animals, hippocampal BDNF levels were increased in both voluntary and involuntary exercise groups compared to controls, yet BDNF was higher in the voluntary condition [67].

While stair use itself can therefore be an opportunity to elevate BDNF in humans as we estimated earlier [25], it is important to distinguish that prospective acute stress can be beneficial for elevating BDNF, yet failing to recover from stress may have adverse effects on BDNF. For instance, it was discussed earlier in this paper that acute stress increases both serum BDNF and cortisol; stress-induced cortisol secretion can accelerate post-stress BDNF decline, suggesting that it can reduce neurogenesis in humans [61]. Therefore, it is important that architectural enrichment not only elevate BDNF through stair use but also facilitate stress recovery, which is proposed through the Neurobiophilia Index that was published recently [26].

### 3.2. Neurobiophilic Architecture and Stress

The stress-recovery element of architectural enrichment is based on the biophilic design literature, which demonstrates that exposure to natural elements reduces salivary cortisol and stress markers [68,69,70,71,72]. The Neurobiophilia Index, which we published earlier [26], expands on those findings by adopting a BDNF- and neurogenesis-centred approach while providing stress recovery. The Index is a quantitative tool that scores indoor architectural environments based on their capacity to enrich the brain through biophilic parameters. In relation to stress recovery, several of its variables have demonstrated restorative effects: natural sounds reduce autonomic stress responses, window green views and indoor plant density support psychophysiological restoration, dynamic sky visibility reduces perceived tension, and temperature within comfortable ranges ranks amongst the least stressful environmental conditions. Notably, several of these same variables extend their influence beyond stress recovery to directly support BDNF and neurogenesis. Daylight at 10,000 lux has been shown to increase hippocampal BDNF and neurogenesis in rodents, whilst olfactory enrichment with novel natural scents promotes neurogenesis in both the hippocampus and olfactory bulb in rodents, and has been associated with significant improvements in cognitive and neural functioning in older adults. Brief passive heat exposure, another index parameter, increases serum BDNF independently of physical activity. Light/dark cycles are also relevant, as disruption through nighttime light exposure suppresses hippocampal BDNF expression. Together, these parameters suggest that the built environment can passively support neurogenesis and BDNF through the same biophilic features that promote stress recovery.

### 3.3. Hypothesised Neurobiological Profiles

The following neurobiological profiles are theoretical predictions derived from the mechanistic relationships established in Section 3.1 and Section 3.2 and are not empirical claims. Their purpose is to generate specific, testable hypotheses about how combinations of individual stress state, neurobiophilic design, and stair use intensity may produce divergent outcomes related to BDNF, neurogenesis, and stress, and to identify which configurations most warrant empirical investigation.

Serum or plasma BDNF, salivary cortisol, and acute stress-recovery markers are the only outcomes in Table 1 that are directly measurable in living humans using established protocols. AHN potential and long-term AHN-dependent stress resilience are inferred outcomes, extrapolated from rodent models and postmortem human evidence through the convergent BDNF-TrkB signalling reviewed in Section 2, and should be interpreted as hypothetical rather than empirical predictions.

Stair use is hypothesised to elevate serum BDNF regardless of the presence of neurobiophilic design. Across all conditions, moderate and short high-intensity stair use (P2, P3, P5, P6, P8, P9, P11, P12) predicts meaningful serum BDNF increases irrespective of whether the building incorporates neurobiophilic features. This reflects the established metabolic basis of peripheral BDNF secretion, which is determined primarily by activity intensity rather than spatial or sensory context. The absence of neurobiophilic design does not negate this benefit; it means cortisol is not concurrently managed and acute stress recovery is not supported, which modulates how effectively that BDNF reaches TrkB receptors and translates into neurogenic outcomes.

Neurobiophilic design, without the confounding effect of passive heat in the index [26], is hypothesised to operate primarily on cortisol and acute stress recovery. Pre-stressed individuals who use stairs without neurobiophilic support can still benefit, but incompletely. Conditions P2 and P3 predict meaningful serum BDNF elevation, and AHN potential is not predicted to be strongly negative, unlike with complete sedentary stress without any architectural enrichment in P1. However, without neurobiophilic support for cortisol management and acute recovery, the downstream neurogenic and long-term resilience benefits remain limited or neutral.

The most adverse condition is P1 and the most favourable is P12. P1, representing a pre-stressed individual with no neurobiophilic design and no stair use, describes the chronic type 2 allostatic overload condition the framework is designed to resolve: cortisol is elevated, BDNF is stable or declining, no recovery is supported, and all long-term neurogenic outcomes are unfavourable. P12, by contrast, places a recovered individual performing short high-intensity stair use within an enriched building with neurobiophilia, where cortisol is moderated, peripheral BDNF is maximally elevated, recovery is supported, and the conditions for TrkB-mediated pro-survival signalling and sustained AHN are hypothesised to be optimal. The full difference between P1 and P12 is attributable to all three factors of the framework operating together, and neither stair use nor neurobiophilic design alone is predicted to reproduce that outcome.

Moderate stair use combined with neurobiophilic design represents the most accessible and sustainable pathway. P11 represents the everyday condition most achievable within typical residential or workplace buildings, combining exercise snacking through stair use with neurobiophilic design features. It predicts meaningful serum BDNF elevation, neutral-to-declining cortisol, supported acute recovery, and favourable long-term neurogenic and resilience outcomes, making it the most scalable and practical configuration of the architectural enrichment framework for the majority of building occupants.

## 4. Future Directions

The architectural enrichment framework presented in this paper is theoretical, and its empirical validation represents a substantive and necessary research agenda. The twelve hypothesised neurobiological profiles in Table 1 are designed to be testable, and the following directions outline how this validation might be pursued across multiple scales, from individual biomarkers to building design and urban infrastructure.

The most immediate priority is the direct empirical testing of the predicted relationships between stair use intensity, neurobiophilic design, stress state, and peripheral biomarkers. Salivary cortisol and serum or plasma BDNF are well-established, validated outcomes that can be measured before and after spatial experiences in real architectural settings. A within-subjects crossover design, in which participants complete multiple conditions of the framework in counterbalanced order, would provide adequate statistical power to detect the interaction effects predicted by Table 1 whilst controlling for individual variability in HPA-axis phenotype and baseline fitness. The contrast between pre-stressed and recovered states could be operationalised using validated acute stress induction protocols prior to spatial exposure, whilst neurobiophilic design quality could be quantified using the Neurobiophilia Index [26].

The framework proposes that voluntariness of stair use is an architecturally determined variable that modulates the cortisol response to activity independently of metabolic demand. Future work should test this prediction directly, examining whether two staircases producing identical exercise intensities yield different cortisol responses depending on occupant-perceived choice and staircase design quality. This would require experimental protocols in which stair design variables, visibility, aesthetics, placement relative to lifts, and wayfinding cues, are systematically varied whilst metabolic demand is held constant through paced ascent protocols. Such research would advance the evidence base for the design of pro-neurogenic buildings considerably beyond what scenario modelling alone can establish [25].

The present framework addresses acute, single-session neurobiological outcomes, but neurosustainability, as a concept, is fundamentally concerned with sustained neurogenic capacity over time [24], which requires longitudinal investigation. Repeated daily exposure to architecturally enriched environments, with the cumulative BDNF stimulation and cortisol management this implies, may produce qualitatively different outcomes from those predicted from acute profiles alone. Future studies should follow participants across months of inhabiting buildings with contrasting Neurobiophilia Index scores, examining changes in BDNF trajectories, cortisol diurnal profiles, hippocampal volume, and stress resilience outcomes. Positive feedback dynamics, in which architectural enrichment progressively improves the neurobiological conditions under which it operates, represent the mechanism through which neurosustainability sustains long-term resilience rather than merely acute recovery.

A potential concern regarding the co-occurrence of neurobiophilic heat exposure and stair use within the same building is whether elevated ambient temperature during physical activity might impair the exercise-induced BDNF response. The available evidence not only dismisses this concern but collectively suggests that heat and exercise may interact beneficially, though the nature of that interaction appears to depend on exercise intensity. Aerobic exercise was found to elevate serum BDNF regardless of environmental temperature across hot (33 °C), cold (7 °C), and moderate (20 °C) conditions, with no significant effect of temperature on the exercise-induced BDNF response [73], suggesting that the metabolic drive to BDNF secretion is sufficiently robust at higher moderate intensities to operate independently of thermal context. By contrast, 180 min of moderate-intensity walking in a hot environment (32 °C wet-bulb globe temperature) produced significant BDNF elevations across young adults, older adults, and individuals with common chronic disease, whilst the same prolonged walking in a temperate condition (16 °C) produced no BDNF change at all [21], suggesting that at lower moderate intensities heat exposure may be necessary rather than merely neutral. Whilst both studies employed moderate-intensity exercise, Collins et al. [73] used a higher moderate-intensity (60% peak wattage) that was independently sufficient to elevate BDNF regardless of temperature, whilst Goulet et al. [21] used a lower moderate intensity at which exercise alone was insufficient in temperate conditions but became sufficient when combined with heat, suggesting that thermal co-stimulation is most consequential at the lower end of the moderate intensity range where the metabolic drive to BDNF secretion is threshold-dependent. Within the architectural enrichment framework, this implies that buildings incorporating heat as a Neurobiophilia Index parameter are unlikely to impair stair-use-induced BDNF under any intensity condition and may augment it specifically in the moderate-intensity exercise snacking scenario most relevant to everyday building use.

Whilst the proposed framework describes a population-level mechanism linking activity-dependent increases in serum BDNF to hippocampal BDNF gene expression and neurogenesis, it is important to acknowledge that individual genetic variability may moderate the magnitude of this effect. The BDNF Val66Met polymorphism, associated with impairment of activity-dependent BDNF secretion [74], represents one such source of inter-individual variability that future empirical work testing this framework should consider. Notably, Val66Met status is rarely reported in the human studies exploring the effect of physical activity on serum or plasma BDNF, representing a broader methodological gap in the field that limits current understanding of whether the proposed pathway operates comparably across genotypes. Future studies seeking to empirically validate this framework are therefore encouraged to incorporate Val66Met genotyping and examine carrier status as a potential moderating variable, particularly in relation to the downstream neurogenic effects proposed here. Beyond genotype, age, sex, fitness level, and baseline allostatic load are all expected to modulate both BDNF and cortisol responses, and future studies should treat these as covariates or stratification variables rather than sources of uncontrolled noise.

Finally, the framework is not limited to any single building type or population. Residential buildings, workplaces, schools, and healthcare settings each present distinct opportunities for architectural enrichment, and future research should examine whether the predicted profiles hold across age groups and clinical populations. Future work should also examine how building-level architectural enrichment articulates with urban design, testing whether enriched buildings embedded in stressful urban environments can meaningfully offset the allostatic burden imposed by the wider context.

## 5. Conclusions

Chronic stress becomes self-perpetuating when adult hippocampal neurogenesis is insufficiently supported, and for most people, this happens in the buildings where they spend 90% of their lives. The same homes, workplaces, and schools that accumulate neurobiological damage could, through architectural enrichment, reverse it. Stair use elevates BDNF. Neurobiophilic design manages cortisol and sustains neurogenesis. Together, they offer what the urban exterior increasingly fails to accomplish: a daily, passive, and chemical stressor-free pathway to stress resilience.

## Figures and Tables

**Table 1:** Hypothesised neurobiological outcome profiles across twelve conditions of the architectural enrichment framework.

Profile	Stress State	Neurobiophilic Design	Stair Use	Serum BDNF †	Salivary Cortisol †	Acute Stress Recovery †	AHN Potential ‡	AHN-Dependent Stress Resilience (Long-Term)
P1	Pre-stressed	Absent	None	→	↑/↑↑	✗	↓	↓
P2	Pre-stressed	Absent	Moderate	↑	↑↑/↑↑↑	✗	→	→
P3	Pre-stressed	Absent	Short HIIT	↑↑	↑↑/↑↑↑	✗	→/↑	→
P4	Pre-stressed	Present	None	→/↑ *	↓	✓	→/↑	→
P5	Pre-stressed	Present	Moderate	↑	→/↑	✓	↑	↑
P6	Pre-stressed	Present	Short HIIT	↑↑	↑	✓	↑	↑
P7	Recovered	Absent	None	→	→	✗	→	→
P8	Recovered	Absent	Moderate	↑	↑	✗	→/↑	→/↑
P9	Recovered	Absent	Short HIIT	↑↑	↑↑	✗	↑	↑
P10	Recovered	Present	None	→/↑ *	↓	✓	↑	↑
P11	Recovered	Present	Moderate	↑	→	✓	↑	↑↑
P12	Recovered	Present	Short HIIT	↑↑	↑	✓	↑↑	↑↑

↑ = hypothesised increase (modest–moderate); ↑↑ = hypothesised substantial increase; ↓ = hypothesised decrease; → = hypothesised negligible change. ✓ = stress recovery supported; ✗ = stress recovery not supported. None = indoor walking or sedentary activity (<3 METs); Moderate = exercise snacking stair use (~3.5–6.0 METs); Short HIIT = high-intensity stair use of approximately 6 min (>6.0 METs), which is estimated to elevate BDNF without the excessive cortisol burden associated with prolonged vigorous activity [25]. * Passive BDNF elevation via neurobiophilic parameters (daylight ≥ 10,000 lux, olfactory enrichment, brief heat exposure) in the absence of stair use. Neurobiophilic design refers to the presence of Neurobiophilia Index parameters [26]. † Peripheral outcomes are directly measurable using established biomarker protocols. ‡ AHN-related outcomes are inferred from rodent and postmortem human evidence and are not directly measurable in living humans; they are presented as hypotheses for future indirect investigation.

## Data Availability

The original contributions presented in this study are included in the article. Further inquiries can be directed to the corresponding author.
